# Functional Interaction between Angiotensin II Receptor Type 1 and Chemokine (C-C Motif) Receptor 2 with Implications for Chronic Kidney Disease

**DOI:** 10.1371/journal.pone.0119803

**Published:** 2015-03-25

**Authors:** Mohammed Akli Ayoub, Yuan Zhang, Robyn S. Kelly, Heng B. See, Elizabeth K. M. Johnstone, Elizabeth A. McCall, James H. Williams, Darren J. Kelly, Kevin D. G. Pfleger

**Affiliations:** 1 Molecular Endocrinology and Pharmacology, Harry Perkins Institute of Medical Research, QEII Medical Centre, Nedlands, Western Australia, Australia; 2 Centre for Medical Research, The University of Western Australia, Crawley, Western Australia, Australia; 3 Department of Medicine, St. Vincent's Hospital, The University of Melbourne, Melbourne, Victoria, Australia; 4 Dimerix Bioscience Limited, Nedlands, Western Australia, Australia; Fondazione IRCCS Ospedale Maggiore Policlinico & Fondazione D’Amico per la Ricerca sulle Malattie Renali, ITALY

## Abstract

Understanding functional interactions between G protein-coupled receptors is of great physiological and pathophysiological importance. Heteromerization provides one important potential mechanism for such interaction between different signalling pathways via macromolecular complex formation. Previous studies suggested a functional interplay between angiotensin II receptor type 1 (AT_1_) and Chemokine (C-C motif) Receptor 2 (CCR2). However the molecular mechanisms are not understood. We investigated AT_1_-CCR2 functional interaction *in vitro* using bioluminescence resonance energy transfer in HEK293 cells and *in vivo* using subtotal-nephrectomized rats as a well-established model for chronic kidney disease. Our data revealed functional heteromers of these receptors resulting in CCR2-Gαi1 coupling being sensitive to AT_1_ activation, as well as apparent enhanced β-arrestin2 recruitment with agonist co-stimulation that is synergistically reversed by combined antagonist treatment. Moreover, we present *in vivo* findings where combined treatment with AT_1_- and CCR2-selective inhibitors was synergistically beneficial in terms of decreasing proteinuria, reducing podocyte loss and preventing renal injury independent of blood pressure in the subtotal-nephrectomized rat model. Our findings further support a role for G protein-coupled receptor functional heteromerization in pathophysiology and provide insights into previous observations indicating the importance of AT_1_-CCR2 functional interaction in inflammation, renal and hypertensive disorders.

## Introduction

The interplay between different hormones, neurotransmitters and chemokines targeting G protein-coupled receptors (GPCRs) has been reported in many cases. To finely integrate signals transduced via different pathways, cells have established various mechanisms of interactions between receptor systems such as functional crosstalk and receptor heteromerization [1]. Heteromerization has been reported for many classes and subtypes of GPCRs, both *in vitro* and *in vivo*, where either a direct or indirect interaction between two different receptors in a macromolecular complex results in one protomer changing the function of another protomer with respect to receptor maturation/trafficking, ligand binding, G protein coupling and/or desensitization/internalization [2]. A receptor heteromer is defined as a “macromolecular complex composed of at least two (functional) receptor units with biochemical properties that are demonstrably different from those of its individual components” [3]. Note that two GPCRs in such a heteromer complex, which is likely to include multiple other proteins, can influence each other’s function without physically touching [4].

The heteromerization concept has evolved rapidly over recent years, bringing more evidence for its importance in physiology and pathology [2]. One important aspect of studying GPCR heteromerization is to investigate whether any of the functional interaction between two hormones/neurotransmitters/chemokines observed *in vivo* could potentially be mediated, at least in part, at the level of their specific receptors. In this context, the functional interaction in the kidney between the receptors for CC chemokine ligand 2 (CCL2; also known as monocyte chemoattractant protein 1 or MCP-1) and angiotensin II (AngII), the main effector peptide of the renin angiotensin system (RAS), constitutes an important model. Indeed, several lines of evidence suggest a relationship between the angiotensin system and the immune system [5–7]. In addition, the link between AngII and CCL2 signalling has been suggested in multiple situations [8–11]. More interestingly, evidence for a potential functional interaction between CCL2 and AngII cognate receptors (CCR2 and AT_1_ receptor, respectively) has only recently emerged, with studies using specific antagonists showing that the combined blockade of the two receptors markedly attenuates renal injury (crescentic glomerulonephritis) [12] and ischemic brain damage [13]. Moreover, a number of studies provide evidence for expression of AT_1_ receptor [14,15] and CCR2 [16,17] in kidney cells, including both podocytes and mesangial cells [15,17]. Indeed overexpression of both of these receptors in podocytes is associated with pathology [16,18]. These findings support our hypothesis that AT_1_ receptor and CCR2 influence each other’s function, with consequent implications for mediating kidney disease progression.

Chronic kidney disease (CKD) is a major cause of morbidity, recurrent hospitalisation and accelerated death, affecting 10–11% of the population in both Europe and the United States [19]. Histopathologically, interstitial inflammatory cell infiltration, cell apoptosis, capillary rarefaction, and fibrosis are the characteristic features of progressive CKD [20]. These structural changes, in turn, result in a loss of glomerular filtration rate (GFR) that is frequently accompanied by progressive proteinuria [20]. The pathological role of AngII has been well documented in the initiation and progression of CKD [21]. Despite current treatments including control of hypertension and blockade of RAS, a considerable proportion of CKD patients continues to progress in association with interstitial macrophage accumulation, suggesting the need for additional immunotherapy [22]. On the other hand, CCL2 has been implicated in the development of a variety of renal diseases including chronic rejection of renal transplantation, lupus nephritis, IgA nephropathy, crescentic glomerulonephritis and diabetic nephropathy by promoting circulating mononuclear cells, as well as tissue macrophage recruitment and activation in the kidney interstitium [23–27]. More importantly, in addition to its role as a mediator of monocyte recruitment, recent studies on both experimental and human diabetic nephropathy have shown that the CCL2/CCR2 system plays a pathological role in the depletion of podocytes and the development of proteinuria [17,28]. Conversely, the blockade of CCL2/CCR2 interaction by either neutralization of CCL2 or CCR2 antagonists has been shown to attenuate progressive kidney damage [29,30].

In this study, we investigated the functional interactions between AT_1_ receptor and CCR2 both *in vitro*, using HEK293FT cells, and *in vivo*, using the sub-total nephrectomized (STNx) rat model characterized by extensive renal mass ablation associated with glomerular RAS upregulation, glomerular hypertension, development of podocyte loss, progressive proteinuria and declining GFR associated with interstitial macrophage infiltration, glomerulosclerosis, and tubulointerstitial fibrosis [31–33]. We carried out *in vitro* experiments to investigate the effect of AT_1_ receptor and CCR2 coexpression on their complex formation, heterotrimeric G protein coupling and β-arrestin2 recruitment. In particular, we utilized the GPCR Heteromer Identification Technology (GPCR-HIT) configuration [4,34–39], the most established version of the Receptor-HIT assay [40] and built upon our recent work assessing receptor-G protein proximity using bioluminescence resonance energy transfer (BRET) [41,42]. Individual and combined treatments with agonists as well as antagonists were performed to investigate the pharmacological profile of AT_1_ receptor-CCR2 complexes. We then investigated *in vivo* whether inhibition of both receptor signalling pathways with a combination of Irbesartan (Irb; AT_1_ receptor antagonist) and Propagermanium (PPG; CCR2 pathway inhibitor) could potentially have a synergistic benefit for CKD treatment, which would be consistent with functional interaction of these receptor signalling pathways.

## Materials and Methods

### Materials

AngII and PPG were from Sigma-Aldrich (Castle Hill, Australia). Irb was from Zhou Fang Pharm Chemical (Shanghai, China). RS504393 was from Tocris. CCL2 was from PeproTech (Rocky Hill, NJ, USA).

### Plasmid Construction

AT_1_ receptor-Rluc8 and CCR2-Rluc8 cDNA constructs were generated from plasmids containing AT_1_ receptor-Rluc and CCR2-Rluc kindly provided by Walter Thomas (University of Queensland) and Aron Chakera (University of Western Australia) respectively. The Rluc coding region was replaced with Rluc8 cDNA from pcDNA3.1-Rluc8 kindly provided by Andreas Loening and Sanjiv Gambhir (Stanford University, CA), as described previously for other GPCR constructs [43]. CCR2-Topaz yellow fluorescent protein (YFP) was also previously generated in the laboratory from CCR2-Rluc. Non-BRET tagged AT_1_ receptor and CCR2 were also provided by Walter Thomas and Aron Chakera respectively. The β-arrestin 2-Venus cDNA construct was prepared previously from pCS2-Venus kindly provided by Atsushi Miyawaki (RIKEN Brain Science Institute, Wako-city, Japan) [43]. Gα_i1_-Rluc8 was generated from the original Gα_i1_-Rluc construct kindly provided by Jean-Philippe Pin and previously reported [41,44]. All constructs were confirmed by DNA sequencing at the Australian Genome Research Facility (Adelaide, Australia).

### Cell culture and transfection

HEK293FT cells were maintained at 37°C, 5% CO_2_ in complete medium (Dulbecco’s modified Eagle’s medium (DMEM) containing 0.3 mg ml^-1^ glutamine, 100 IU ml^-1^ penicillin, and 100 μg ml^-1^ streptomycin) (Gibco BRL, Carlsbad, CA) supplemented with 10% fetal calf serum (FCS; Gibco). Transient transfections were carried out using GeneJuice (Merck, Kilsyth, Australia) or FuGENE (Promega, Alexandria, Australia) according to the manufacturer’s instructions and the experiments were performed 48 hours post-transfection.

### GPCR-HIT BRET assays

HEK293FT cells were transfected as specified in the figure legends using GeneJuice (Merck, Kilsyth, Australia) or FuGENE (Promega, Alexandria, Australia) as per the manufacturer’s instructions. 48 h post-transfection, BRET measurements were carried out in white 96-well plates using coelenterazine h (5 μM final), or following incubation at 37°C, 5% CO_2_ for 2 h with 30 μM EnduRen (Promega, Alexandria, Australia) for assessing arrestin recruitment kinetics, as described previously [4]. BRET detection was carried out in live cells at 37°C by measuring sequential light emissions at 400–475 nm and 520–540 nm using the VICTOR Light plate reader with Wallac 1420 software (PerkinElmer, Melbourne, Australia). The ligand-induced BRET signal was calculated by subtracting the ratio of 520–540 nm emission over 400–475 nm emission for a vehicle-treated cell sample from the same ratio for a second aliquot of the same cells treated with ligand (ligand-induced BRET), as described previously [43]. With kinetic data, the final pre-treatment measurement is presented at the zero timepoint (time of ligand or vehicle addition).

### Measurement of inositol-1-phosphate (IP_1_) production

The determination of IP_1_ accumulation was performed using the IP-One HTRF assay (CisBio Bioassays, Bagnol sur Ceze, France), as described previously [45]. Briefly, cells were transfected and seeded into white 96-well plates. 48 h post-transfection cell media was replaced with 50 μl stimulation buffer containing agonists as indicated. After a 30 min incubation at 37°C, 5% CO_2_, cells were lysed with 12.5 μl of the supplied conjugate-lysis buffer containing d2-labeled IP_1_. This was immediately followed by addition of 12.5 μl of conjugate-lysis buffer containing terbium cryptate-labeled anti-IP_1_ antibody. Following a 1 h incubation at room temperature, fluorescence was measured at 620 and 665 nm 50 μs after excitation at 337 nm using an EnVision 2102 plate reader (PerkinElmer).

### Animal Ethics Statement

All animal experiments were conducted with approval from the St Vincent’s Hospital Animal Ethics Committee (AEC) in accord with the National Health and Medical Research Council Australian Code of Practice for the Care and Use of Animals for Scientific Purposes. The approved AEC code was 005/12.

### Animal experimental design and surgery

Six week old, male Sprague-Dawley (SD) rats weighing 200–250g were sourced from the Animal Resources Centre (Western Australia). All rats received normal rat chow (Certified Rodent Diet #5002, LabDiet, USA) and drinking water ad libitum. All animals were housed in a stable environment maintained at 22 ± 1°C with a 12-hour light/dark cycle commencing at 6am. STNx surgery was performed in the operating theatre at St Vincent’s Experimental Surgical Unit. All surgical procedures were performed as previously published [46].

One hundred rats were randomized to 5 groups of 20 animals each. Anaesthesia was achieved with 3% isoflurane/97% oxygen in a tidal volume of 1 ml 100g^-1^ body weight. The control group (n = 20) underwent sham surgery consisting of laparotomy and manipulation of both kidneys before wound closure. The other 80 rats underwent STNx performed by right subcapsular nephrectomy and infarction of approximately 2/3 of the left kidney by selective ligation of two out of the three extrarenal branches of the left renal artery [47]. Any pain experienced as a result of the surgical procedure performed on these animals was minimized with the use of buprenorphine (0.03mg/kg) directly following surgery as routine. In the event of unrelieveable pain, the rats were euthanased with an overdose of Lethabarb (Sodium Pentobarbitone, 120 mg/kg). Two weeks post-surgery, STNx animals were then randomly assigned to 4 groups to receive treatment with either PPG (30 mg kg^-1^ day^-1^ gavaged) or Irb (10 mg kg^-1^ day^-1^, in drinking water) or combination of Irb (10 mg kg^-1^ day^-1^, in drinking water) and PPG (30 mg kg^-1^ day^-1^ gavaged), or vehicle (1% CMC) for 12 weeks. Every 4 weeks, rats were weighed and systolic blood pressure (SBP) was determined in preheated conscious rats via tail-cuff plethysmography using a non-invasive blood pressure (NIBP) controller and Powerlab (AD instruments, Bella Vista, Australia). Urine was collected over 24 hours at the end of the study for subsequent urinary biochemistry analysis.

### Proteinuria

Proteinuria was determined from an aliquot of urine collected during the 24 hour period in the metabolic cages. In brief, once thawed, 20μl of urine was added to a clinical uristix (Bayer Diagnostics Manufacturing, Sudbury, England) to obtain an approximate urine protein concentration. Dilutions were made with 0.9% saline and samples were assayed on a Cobas Integra 400 employing the Tina-Quant-Albu2 assay as previously described [48]. The method was modified by calibrating the analyser with rat albumin standards (Sigma-Aldrich, Missouri, USA). Proteinuria was expressed as mg day^-1^ using the total volume of urine collected over the 24 hours.

### Glomerular filtration rate (GFR)

GFR was determined by injecting a single shot of 99Tc-DTPA into the tail vein of the rats. Blood was sampled after 43 min as previously described [49] and expressed as ml min^-1^.

### Histopathology

At the end of the study, rats were anaesthetised (Nembutal 60 mg kg^-1^ body wt i.p.; Boehringer-Ingelheim, North Ryde, Australia). Kidneys were excised, de-capsulated and sliced transversely. Half of the kidney was snap-frozen for molecular biology and the other half was immersion fixed with formalin and paraffin-embedded for subsequent light microscopic evaluation. Histopathological changes such as glomerulosclerosis and tubulointerstitial fibrosis in the kidney were assessed in a masked protocol. Sections were stained with either periodic acid Schiff’s stain (PAS) for glomerulosclerosis or Masson’s modified trichrome to demonstrate collagenous matrix [46].

### Immunohistochemistry

Immunohistochemical staining was performed on 4 μm tissue sections as previously described [50]. Sections were dewaxed in histolene, hydrated through graded ethanols, and then immersed in tap water. The antigen retrieval involved heating sections in a pressure cooker in 10mM sodium citrate buffer (pH 6) for 4 min, and allowing them to cool at room temperature for 30 min. To block non-specific staining due to endogenous peroxidase activity, all sections were incubated with 3% hydrogen peroxidase for 10 min at room temperature, followed by 3 times of 5 min wash with Phosphate Buffered Saline (PBS) before being incubated for 20 min with normal goat serum (NGS) diluted 1:10 with PBS, pH 7.4 as protein block. Sections were then incubated with rabbit anti-WT-1 antibody (Santa Cruz, 1:400 diluted with PBS) or mouse anti rat ED-1 (serotec, 1:300 diluted with PBS) at 4°C for 18 hours. The following day, sections were thoroughly washed in PBS (3 x 5 min), and then incubated with goat anti-rabbit or anti-mouse HRP (DAKO, CA) for 30 min at room temperature. Localization of the peroxidase conjugates was achieved using 3,3’-diaminobenizidine tetrahydrochloride (DAB; DAKO, CA) as a chromogen, for 1–3 min (development time assessed with light microscope), slides were then rinsed in tap water for 5 min to stop the development, counterstained in Mayer’s haemotoxylin, differentiated in Scott’s tap water, dehydrated, cleared and mounted in DPX. Sections incubated with 1:10 NGS, instead of the primary antiserum, served as negative controls.

### Glomerulosclerotic index

In 4 μm kidney sections stained with PAS, 50 glomeruli from each rat were examined in a masked protocol. The extent of sclerosis in each glomerulus was subjectively graded on a scale of 0 to 4, as previously described [51] with Grade 0, normal; Grade 1, sclerotic area up to 25% (minimal); Grade 2, sclerotic area 25–50% (moderate); Grade 3, sclerotic area 50–75% (moderate to severe) and Grade 4, sclerotic area 75–100% (severe). A glomerulosclerotic index (GSI) was then calculated using the formula:

$$GSI=\overset{4}{\underset{i=0}{\sum }}Fi\left(i\right)$$

where Fi is the % of glomeruli in the rat with a given score (i).

### Quantitation of matrix deposition

To measure interstitial fibrosis in the kidney, 10 random non-overlapping fields from 10 rats per group were captured and digitised using a Carl Zeiss microscope attached to AxioCamMRc5 digital camera (Carl Zeiss, North Ryde, Australia) under 200x magnification. Digital images were then loaded onto a Pentium D Dell computer. An area of blue in the cortex of the kidney was selected for its colour range and the proportional area of the selected colour range was then quantified using image analysis (AxioVision Release 4.8.1; Carl Zeiss, North Ryde, Australia) based on the method adapted from Lehr et al [52]. Data were expressed as percentage change per area [46].

### Quantitation of podocytes and macrophages

Quantitation of podocytes was assessed by examining approximately 20–30 hilar glomeruli per animal with a light microscope at x400, expressed as numbers per glomerular cross section (gcs). Macrophages were counted by examining 5 fields per section with a light microscope at x200, expressed as numbers per area.

### Data analysis and statistical procedures

All *in vitro* data were analysed using Prism software (GraphPad, San Diego, CA, USA). Dose-response curves were fitted using nonlinear regression and statistical significance was determined by ANOVA with Bonferroni post-test or unpaired t-test where appropriate. For *in vivo* data, analysis was performed using Statview II + Graphics package (Abacus Concepts, Berkeley, CA). Statistical significance was determined by one-way ANOVA with Fishers post-hoc comparison. Where data were not normally distributed, statistical analysis was carried out following logarithmic transformation. A *p*-value < 0.05 was regarded as statistically significant.

## Results

### GPCR-HIT assay—AT_1_ receptor activation negatively modulates CCR2-Gα_i1_ coupling

**Fig 1:**
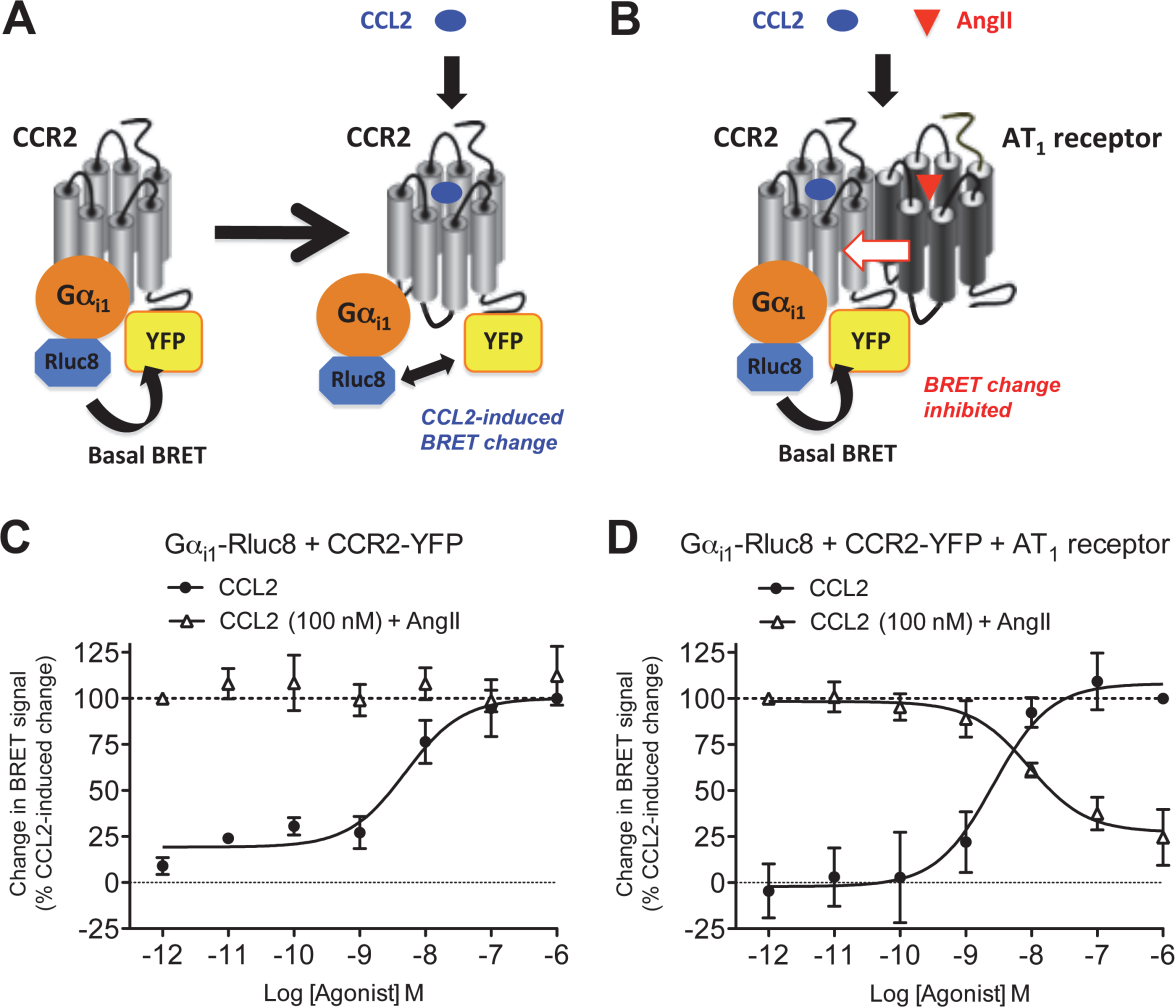
Use of GPCR-HIT to show effect of AT_1_ receptor activation on CCR2/Gα_i1_ proximity. Live HEK293FT cells co-expressing Gα_i1_-Rluc8 with either CCR2-YFP only (A and C) or CCR2-YFP and AT_1_ receptor (B and D) were used to measure the change in BRET signal with increasing doses of CCL2, or 100 nM CCL2 with increasing doses of AngII (C and D). It has been observed previously [39,41,42,44] that the BRET signal can increase or decrease as a consequence of the ligand-induced conformational change associated with Gα_i1_ protein activation (A). The data are presented here as change in BRET signal as a percentage of CCL2-induced change for ease of interpretation. 100% is defined in each individual experiment as the decrease in BRET signal observed upon addition of 1 μM CCL2 (for curves with black circles), or 100 nM CCL2 + 1 pM AngII (for curves with white triangles). Data are presented as mean ± SEM of three (C) or five (D) independent experiments. CCL2 logEC_50_ in (C) = -8.27 ± 0.20 and in (D) = -8.67 ± 0.13 (not significantly different (unpaired t-test, P > 0.05)). In the presence of AT_1_ receptor, logIC_50_ for AngII inhibiting 100 nM CCL2 = -8.19 ± 0.31.

CCR2 primarily signals via coupling to the inhibitory Gα_i_ protein. We therefore utilized the BRET assay to monitor agonist-promoted conformational changes within the complex formed by Rluc8-tagged Gα_i1_ (Gα_i1_-Rluc8) and YFP-tagged CCR2 (Fig. 1A) indicative of G protein activation as shown previously [39,41,42,44]. For the putative functional interaction between AT_1_ receptor and CCR2, we used the GPCR-HIT assay on the BRET platform (Fig. 1B). This is an assay configuration whereby one receptor (eg. CCR2) is labelled with one component (eg. YFP) of a proximity-based reporter system (eg. BRET), the complementary component of which (eg. Rluc8) is fused to a receptor interacting partner (eg. Gα_i1_). Treatment with a ligand (eg. AngII) selective for the untagged receptor (eg. AT_1_ receptor) results in modulation of the proximity of the tagged receptor and the interacting partner, resulting in a change in BRET signal that is indicative of functional interaction between the two receptors [4,40].

In cells co-expressing Gα_i1_-Rluc8 and CCR2-YFP, CCL2 resulted in dose-dependent Gα_i1_ activation as expected, reaching maximal activation by 100 nM CCL2 (Fig. 1C). Furthermore, increasing doses of AngII had no effect on the change in BRET signal induced by 100 nM CCL2 in the absence of AT_1_ receptor (Fig. 1C). These findings indicate that AngII is not acting directly on CCR2 to mediate its effects, as it requires the presence of the AT_1_ receptor. In cells co-expressing Gα_i1_-Rluc8, CCR2-YFP and AT_1_ receptor, a similar dose-dependent change in BRET signal was induced by CCL2. However in contrast, the Gα_i1_ protein activation induced by 100 nM CCL2 was inhibited by AngII in a dose-dependent manner (Fig. 1D). This AngII-dependent modulation provides evidence for a functional interaction between CCR2 and AT_1_ receptor.

### Lack of synergy with respect to inositol phosphate signalling

**Fig 2:**
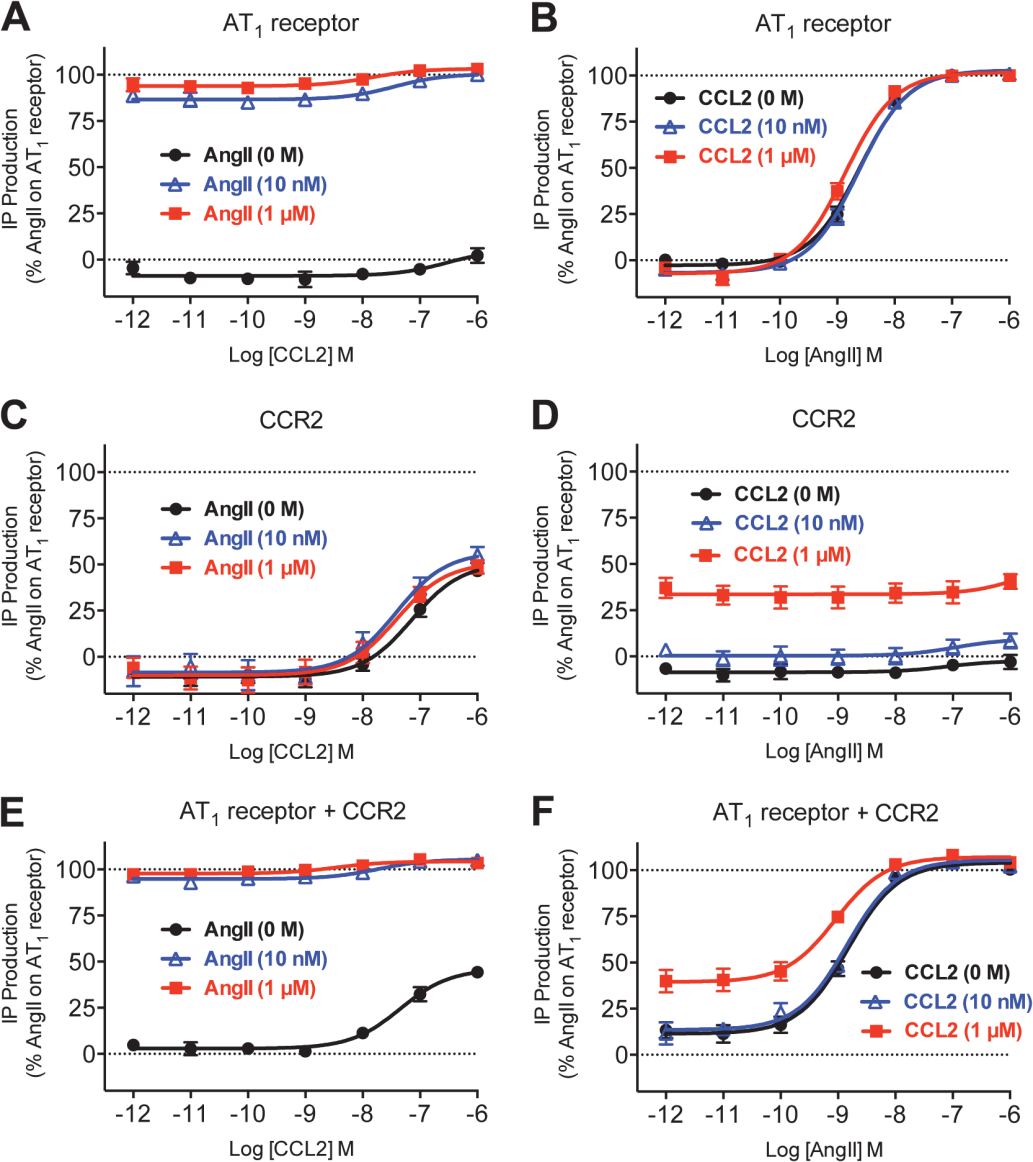
Measurement of inositol-1-phosphate (IP_1_) in cells co-expressing AT_1_ receptor and CCR2. HEK293FT cells expressing AT_1_ receptor (A and B), CCR2 (C and D) or both AT_1_ receptor and CCR2 (E and F) were used to measure agonist-induced IP_1_ production after 30 min at 37°C with increasing doses of CCL2 in the presence of 0, 10 or 1000 nM AngII (A, C and E) or with increasing doses of AngII in the presence of 0, 10 or 1000 nM CCL2 (B, D and F). Data are shown as a percentage of AngII-induced IP_1_ production in cells expressing AT_1_ receptor alone. Data are presented as mean ± SEM of five independent experiments. LogEC_50_ values are shown in Table 1.

AT_1_ receptor primarily signals through Gα_q/11_, leading to increases in inositol phosphate signalling. Furthermore, CCR2 has been shown to couple to Ca^2+^ signalling through both pertussis toxin sensitive [39,53] and insensitive [39] mechanisms. We observed strong and potent AngII-induced IP_1_ production with AT_1_ receptor (Fig. 2A and B; Table 1), as well as weaker and less potent CCL2-induced IP_1_ production with CCR2 (Fig. 2C and D; Table 1). However, no discernible synergistic effect was observed as a consequence of AT_1_ receptor and CCR2 co-expression (Fig. 2E and F; Table 1).

**Table 1:** LogEC_50_ data for IP_1_ production shown in Fig. 2.

	[AngII] (nM)	CCL2 LogEC_50_	[CCL2] (nM)	AngII LogEC_50_
**AT**_**1**_ **receptor**	**0**	ND	**0**	−8.61 ± 0.04
	**10**	ND	**10**	−8.65 ± 0.04
	**1000**	ND	**1000**	−8.85 ± 0.06
**CCR2**	**0**	−7.13 ± 0.06	**0**	ND
	**10**	−7.37 ± 0.09	**10**	ND
	**1000**	−7.41 ± 0.03	**1000**	ND
**AT**_**1**_ **receptor + CCR2**	**0**	−7.31 ± 0.07	**0**	−8.81 ± 0.06
	**10**	ND	**10**	−8.86 ± 0.07
	**1000**	ND	**1000**	−9.02 ± 0.06

Data are mean ± SEM, n = 5.

Note: co-treatment with 10 or 1000 nM AngII did not significantly alter the CCL2 logEC_50_ with cells expressing CCR2 and likewise, co-treatment with 10 or 1000 nM CCL2 did not significantly alter the AngII logEC_50_ with cells expressing either AT_1_ receptor or both AT_1_ receptor and CCR2 (P > 0.05).

In cells expressing AT_1_ receptor and CCR2, a significant difference was observed between the CCL2 logEC_50_ and AngII logEC_50_ in the absence of the other ligand (P < 0.05).

ND, not determined.

### GPCR-HIT assay—Evidence for potentiation of β-arrestin2 recruitment as a consequence of AT_1_ receptor-CCR2 heteromerization

**Fig 3:**
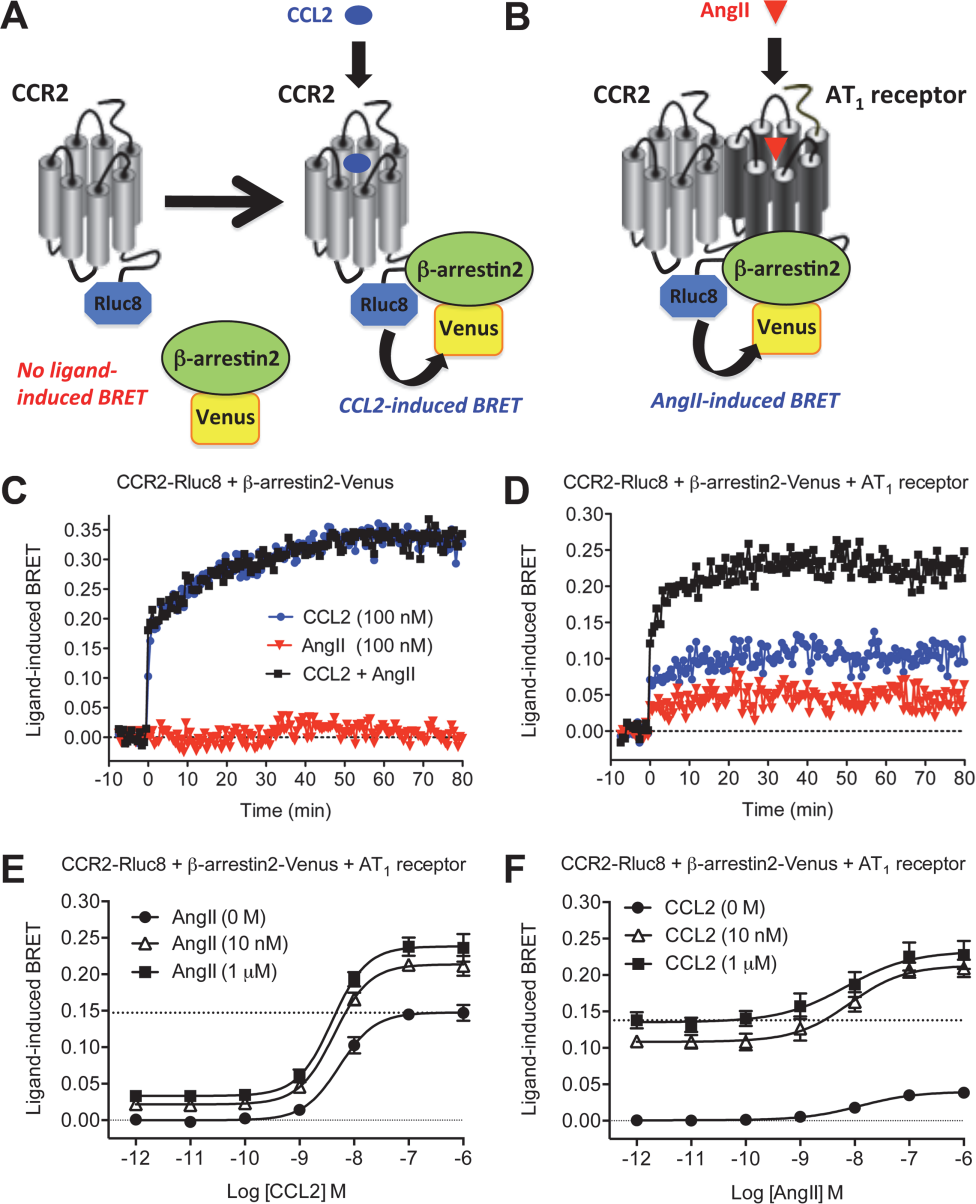
GPCR-HIT analysis to show effect of AT_1_ receptor activation on CCR2/β-arrestin2 proximity. CCL2-induced activation of CCR2-Rluc8 leads to recruitment of β-arrestin2-Venus, resulting in a BRET signal (A). In the presence of AT_1_ receptor, AngII induces recruitment of β-arrestin2-Venus to the AT_1_-CCR2 heteromer, again resulting in a BRET signal (B) and thereby providing evidence for receptor heteromerization. Live HEK293FT cells co-expressing CCR2-Rluc8 and β-arrestin2-Venus in the absence (C) or presence (D) of AT_1_ receptor were used to measure the increase in agonist-induced BRET signal in real-time at 37°C before and after stimulation with 100 nM CCL2, AngII or both simultaneously. Data in (C) and (D) are representative of three independent experiments. Live HEK293FT cells co-expressing CCR2-Rluc8, β-arrestin2-Venus and AT_1_ receptor were then used to measure the increase in agonist-induced BRET signal after 30 min at 37°C with increasing doses of CCL2 in the presence of 0, 10 or 1000 nM AngII (E) or with increasing doses of AngII in the presence of 0, 10 or 1000 nM CCL2 (F). Data in (E) and (F) are presented as mean ± SEM of five independent experiments. LogEC_50_ values are shown in Table 2.

GPCR-HIT assays were performed in real-time on HEK293FT cells co-expressing CCR2-Rluc8 and β-arrestin2-Venus in the absence (Fig. 3A and C) or presence (Fig. 3B and D) of AT_1_ receptor. Treatment with CCL2 caused recruitment of β-arrestin2-Venus to CCR2-Rluc8 in a manner that was not affected by AngII co-treatment in the absence of AT_1_ receptor (Fig. 3C). Furthermore, no AngII-induced BRET signal was observed. This again indicates that AngII is not acting directly on CCR2 to mediate its effects, as it requires the presence of the AT_1_ receptor.

In contrast, in the presence of AT_1_ receptor, AngII induced a BRET signal indicative of recruiting β-arrestin2-Venus proximal to CCR2-Rluc8 (Fig. 3D). This effect is not observed simply because β-arrestin2-Venus is translocated to the plasma membrane upon activation of AT_1_ receptor. If this was the case, a similar response would be expected if CCR2 was substituted with any GPCR located in the plasma membrane. We have previously published that AngII treatment of HEK293FT cells expressing bradykinin receptor 2-Rluc8, β-arrestin2-Venus and AT_1_ receptor did not result in an increase in BRET signal, even though AT_1_ receptor was expressed functionally at the plasma membrane and addition of bradykinin resulted in a very robust BRET signal [4].

Remarkably, co-treatment with CCL2 and AngII resulted in a signal that was more than additive (Fig. 3D). This effect was also illustrated by the dose-response data, where co-stimulation with AngII (10 nM or 1 μM) increased the maximal CCL2-induced BRET signal to an extent that was more than additive (Fig. 3E) without significantly altering potency (Table 2). Similarly, AngII induced a dose-dependent BRET increase and the co-stimulation with CCL2 (10 nM or 1 μM) increased the maximal response to an extent that was more than additive (Fig. 3F), and again without altering potency (Table 2).

**Table 2:** LogEC_50_ data for CCR2/β-arrestin2 proximity shown in Fig. 3.

	[AngII] (nM)	CCL2 LogEC_50_	[CCL2] (nM)	AngII LogEC_50_
**CCR2-Rluc8 +**	**0**	−8.26 ± 0.11	**0**	−8.05 ± 0.25
β-**arrestin2-Venus +**	**10**	−8.35 ± 0.10	**10**	−8.29 ± 0.31
**AT**_**1**_ **receptor**	**1000**	−8.40 ± 0.08	**1000**	−8.30 ± 0.36

Data are mean ± SEM, n = 5.

Note: no significant difference was observed between values (P > 0.05).

### Effect of the combined AT_1_ receptor and CCR2 antagonists on β-arrestin2 recruitment

**Fig 4:**
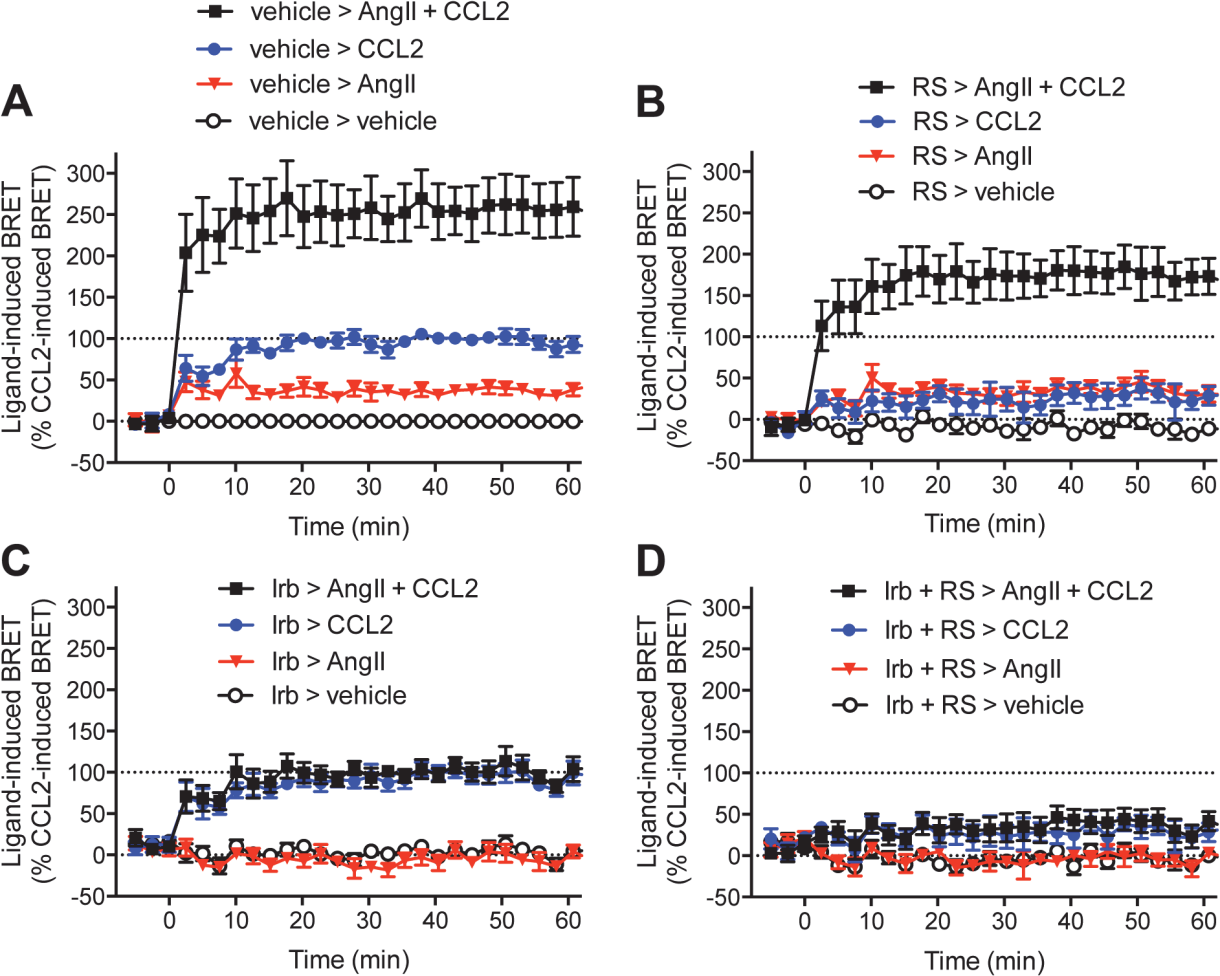
Effect of combined AT_1_ receptor and CCR2 blockade on GPCR-HIT with β-arrestin2. Real-time kinetic profiles were generated with live HEK293FT cells co-expressing CCR2-Rluc8, β-arrestin2-Venus and AT_1_ receptor with 30 min preincubation at 37°C with vehicle (A), RS504393 (RS; 10 μM; B), Irbesartan (Irb; 10 μM; C), or both combined (D). Cells were then stimulated with AngII and/or CCL2 (100 nM) and BRET signals measured. 100% is defined as the mean increase in BRET signal observed 20 min after addition of CCL2 and following preincubation with vehicle. Data are presented as mean ± SEM of five independent experiments.

We used AT_1_ receptor-selective (Irb) and CCR2-selective (RS504393) antagonists to assess the impact of combined receptor blockade on β-arrestin2 interaction with AT_1_ receptor-CCR2 complexes (Fig. 4). In vehicle pre-treated cells, AngII and CCL2 induced BRET increases with a more than additive effect when applied together (Fig. 4A), consistent with Fig. 3D, E and F. Pre-treatment with 10 μM RS504393 decreased the CCL2-dependent signal and the CCL2 component of the co-stimulation (Fig. 4B), indicative of partial blockade of CCL2-induced β-arrestin2 recruitment by this compound. In contrast, pre-treatment with 10 μM Irb totally abolished both the AngII-dependent signal and the AngII component of the co-stimulation (Fig. 4C). When both antagonists were simultaneously applied, there was a dramatic inhibition of β-arrestin2 recruitment mediated by either individual or simultaneous AngII and CCL2 stimulation (Fig. 4D). These data confirm the specificity of the CCL2 and AngII effects, and provide justification for testing a combination of inhibitors *in vivo*.

### CKD Animal characteristics

**Fig 5:**
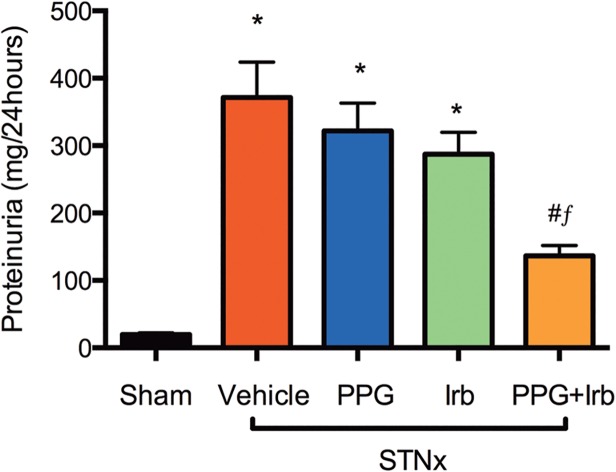
Proteinuria in STNx rats. STNx rats developed proteinuria of a level more than an order of magnitude higher than sham. In contrast to PPG or Irb monotherapies, treatment with PPG+Irb was associated with a significant reduction in proteinuria. Data are expressed as mean ± SEM. *, P <0.05 vs sham; #, P <0.05 vs vehicle-treated; *f*, P <0.05 vs Irb-treated STNx rats. Animal numbers: Sham = 20, STNx = 19, STNx+PPG = 17, STNx+Irb = 19 and STNx+PPG+Irb = 16.

We performed *in vivo* studies using the STNx model of progressive kidney disease where animals were treated or not with either PPG, Irb or both combined (PPG+Irb). In comparison with sham animals, STNx rats developed hypertension (Table 3) and dysregulation of renal functions characterized by a decline in GFR (Table 3) and an increase in proteinuria (Fig. 5). Although not resulting in a significant reduction in blood pressure (Table 3), combined treatment (PPG+Irb) was associated with a significant reduction in proteinuria (Fig. 5) compared to vehicle, in contrast to Irb or PPG monotherapies. Furthermore, proteinuria with PPG+Irb was significantly lower than with Irb treatment alone (Fig. 5).

**Table 3:** Animal characteristics.

Group	BW (g)	SBP (mmHg)	GFR (ml min^-1^)
**Sham**	532 ± 17	145 ± 6^#^	5.09 ± 0.26^#^
**STNx**	509 ± 13	220 ± 7*	0.43 ± 0.14*
**STNx + PPG**	490 ± 30	247 ± 13*	0.49 ± 0.31*
**STNx + Irb**	493 ± 15	180 ± 6^#^	0.92 ± 0.13* ^#^
**STNx + PPG + Irb**	478 ± 7	213 ± 9*	0.90 ± 0.13* ^#^

*, P< 0.05 vs Sham;

^#^, P<0.05 vs STNx

STNx, Subtotal nephrectomized; Irb, Irbesartan; PPG, Propagermanium

Animal numbers: Sham = 20, STNx = 19, STNx+PPG = 17, STNx+Irb = 19 and STNx+PPG+Irb = 16. Data are mean ± SEM.

### Macrophage Infiltration

**Fig 6:**
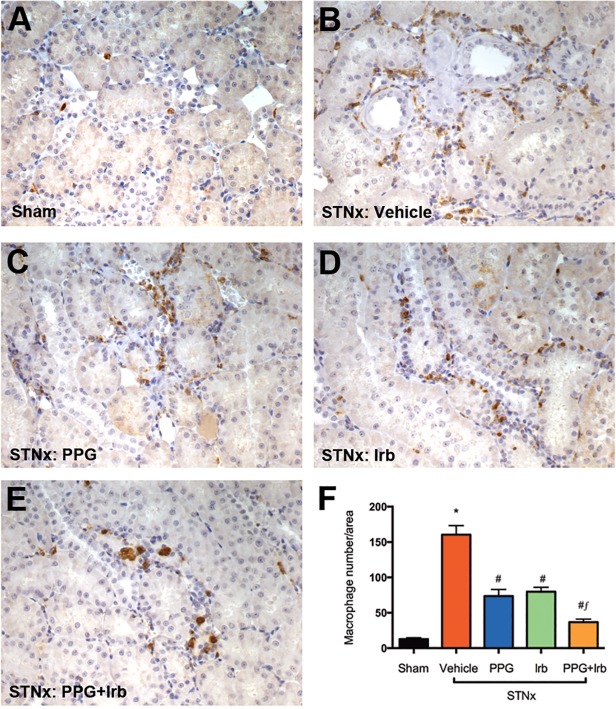
ED-1 (macrophage) staining from STNx rats. As illustrated by representative photomicrographs, in sham rats (A), only occasional macrophages were observed in the interstitium, while STNx rats (B) were associated with numerous macrophages. When compared to PPG (C) and Irb (D) mono-therapy, treatment of STNx animals with PPG+Irb (E) was associated with a further reduction in the number of macrophages. Magnification x200. Quantitative data (F) are expressed as mean ± SEM. *, P <0.05 vs sham; #, P <0.05 vs vehicle-treated;*f*, P <0.05 vs Irb-treated STNx rats. Animal numbers: Sham = 20, STNx = 19, STNx+PPG = 17, STNx+Irb = 19 and STNx+PPG+Irb = 16.

The influx of infiltrating macrophages has been consistently implicated in cell apoptosis, proteinuria and interstitial fibrosis in CKD [54]. As shown in Fig. 6B and F, immunostaining with a macrophage marker ED-1 in STNx rats demonstrated a significant increase in the number of macrophages when compared with sham (Fig. 6A and F). Combined treatment (PPG+Irb) of STNx rats was associated with a further reduction in macrophage infiltration (Fig. 6E and F) when compared to PPG (Fig. 6C and F) or Irb (Fig. 6D and F) monotherapy.

### Podocyte loss

**Fig 7:**
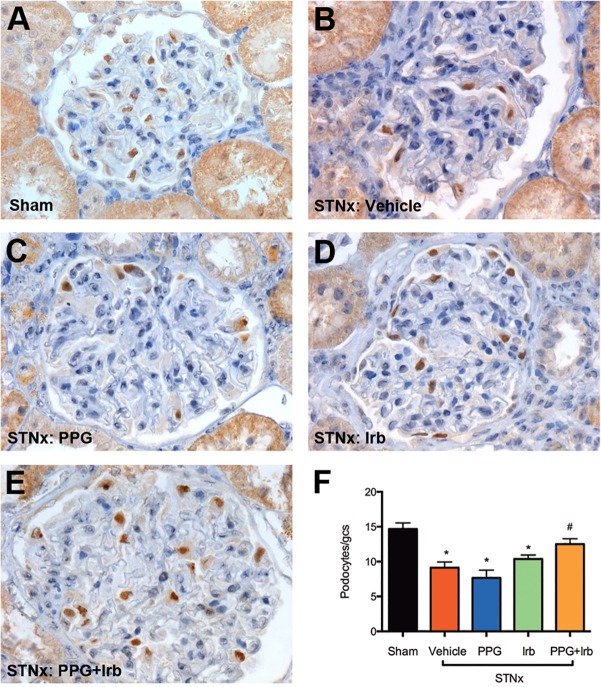
WT-1 (podocyte) staining from STNx rats. As illustrated by representative photomicrographs, in comparison with sham rats (A), STNx rats (B) were associated with a significant increase in podocyte loss. Treatment of STNx rats with either PPG (C) or Irb (D) alone did not affect podocyte loss significantly, whereas treatment with PPG+Irb (E) was associated with reduced podocyte loss. Magnification x400. Quantitative data (F) are expressed as mean ± SEM. *, P <0.05 vs sham; #, P <0.05 vs vehicle-treated STNx rats. Animal numbers: Sham = 20, STNx = 19, STNx+PPG = 17, STNx+Irb = 19 and STNx+PPG+Irb = 16.

Podocyte loss has been implicated in the pathogenesis of proteinuria in CKD. WT-1 (podocyte marker) immunostaining in STNx rats demonstrated a significant reduction in the number of podocytes (Fig. 7B and F) when compared with sham (Fig. 7A and F). Treatment of STNx rats with PPG in combination with Irb significantly attenuated podocyte loss (Fig. 7E and F) when compared to vehicle (Fig. 7B and F), in contrast to PPG (Fig. 7C and F) or Irb (Fig. 7D and F) monotherapy.

### Fibrosis

**Fig 8:**
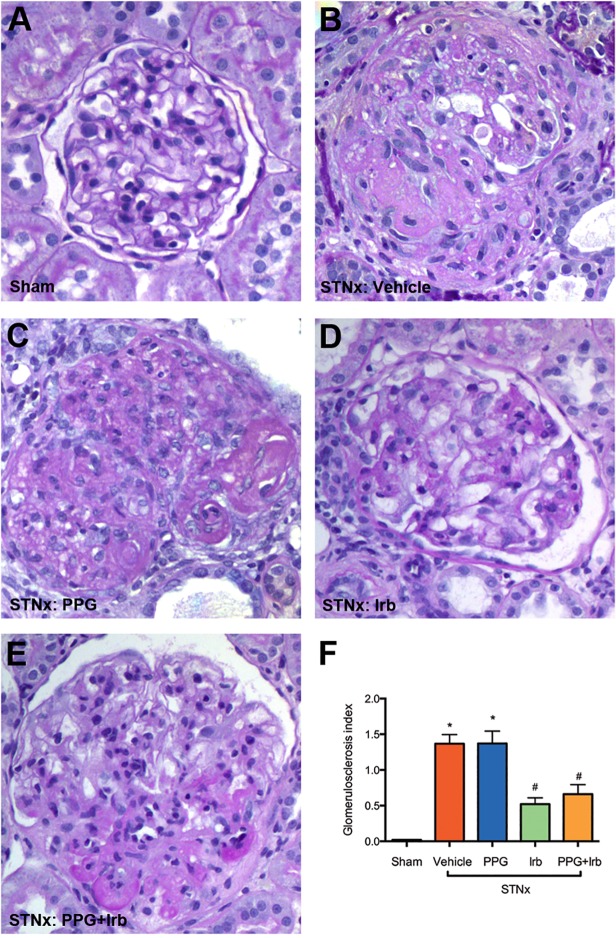
Glomerulosclerosis in STNx rats. As illustrated by representative photomicrographs, in sham rats (A) there was minimal glomerulosclerosis as determined by PAS stain, while STNx rats (B) demonstrated severe glomerulosclerosis. Intervention with PPG alone in STNx rats had no effect on reducing glomerulosclerosis (C). Treatment of STNx rats with Irb (D) or a combination of PPG+Irb (E) was associated with a significant reduction in glomerulosclerosis when compared to vehicle-treated STNx rats (B). Magnification x400. Quantitative data (F) are expressed as mean ± SEM. *, P <0.05 vs sham; #, P <0.05 vs vehicle-treated STNx rats. Animal numbers: Sham = 20, STNx = 19, STNx+PPG = 17, STNx+Irb = 19 and STNx+PPG+Irb = 16.

**Fig 9:**
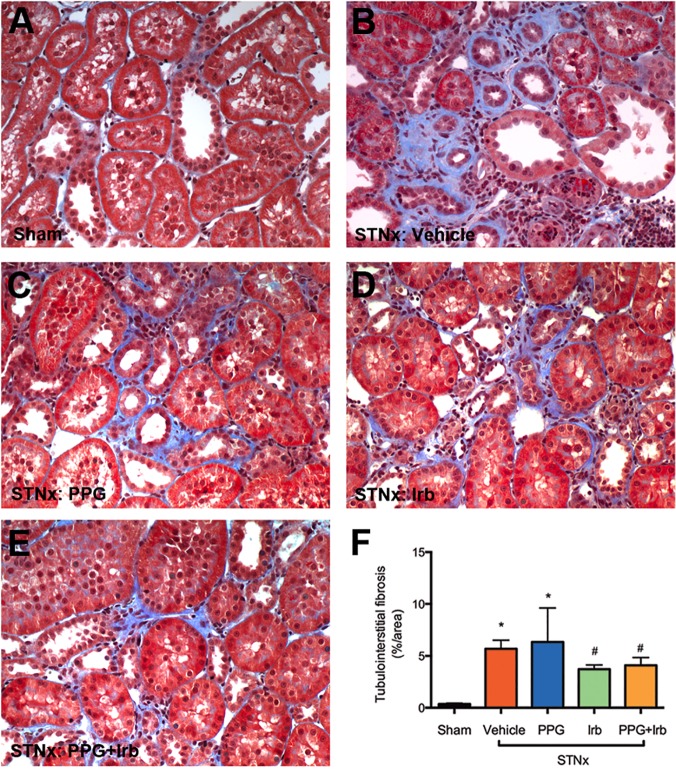
Tubulointerstitial fibrosis in STNx rats. As illustrated by representative photomicrographs, in sham rats (A) there was minimal cortical tubulointerstitial fibrosis as determined by Masson’s trichrome staining, while STNx rats (B) were associated with a significant increase in tubulointerstitial fibrosis (blue). PPG (C) did not reduce this fibrosis, whereas treatment of STNx rats with Irb (D) or PPG+Irb (E) was associated with a significant reduction. Magnification x200. Quantitative data (F) are expressed as mean ± SEM. *, P <0.05 vs sham; #, P <0.05 vs vehicle-treated STNx rats. Animal numbers: Sham = 20, STNx = 19, STNx+PPG = 17, STNx+Irb = 19 and STNx+PPG+Irb = 16.

Glomerulosclerosis and tubulointerstitial fibrosis are prominent features in CKD. STNx rats developed severe glomerulosclerosis (Fig. 8B) and tubulointerstitial fibrosis (Fig. 9B) compared to sham (Fig. 8A and 9A). Irb but not PPG significantly attenuated glomerulosclerosis (Fig. 8C, D and F) and tubulointerstitial fibrosis (Fig. 9C, D and F) when compared to vehicle treated STNx rats (Fig. 8F and 9F). The combination treatment (PPG+Irb) had a similar effect to Irb alone (Fig. 8E, 8F, 9E and 9F).

## Discussion and Conclusions

Through the application of the GPCR-HIT assay [4], configured on the BRET platform and utilising both Gα_i1_ protein and β-arrestin2 as interacting partners, we have generated evidence consistent with a functional interaction between AT_1_ receptor and CCR2 at the receptor level in HEK293FT cells. Very interestingly, AT_1_ receptor activation appears to inhibit the conformational change associated with CCR2-Gα_i1_ coupling. We suggest two potential explanations for this effect: There may be a direct allosteric modulation of CCR2 by the activated (AngII-bound) AT_1_ receptor through a macromolecular complex, resulting in modulation of the receptor-Gα_i1_ conformational change. Alternatively, co-activation of both receptors may result in an increase in β-arrestin recruitment that switches off CCR2-Gα_i1_ signalling more robustly. Furthermore, there could be a combination of both of these effects as they are not mutually-exclusive.

The functional interaction of these receptors is also supported by the BRET signals observed when monitoring β-arrestin2 recruitment proximal to CCR2 in the presence of AT_1_ receptor, where combined treatment with CCL2 and AngII induced a substantially higher BRET signal than observed with CCL2 or AngII alone (more than additive). Indeed this is consistent with our previous findings for CCR2-CCR5 and CCR2-CXCR4 heteromers [4]. An interesting question raised by our results is whether the conformation of the Gα_i1_ in the complex prior to addition of CCL2 somehow causes impaired recruitment of β-arrestin2 to the AT_1_ receptor, perhaps as a consequence of the heterotrimeric G protein complex straddling the intracellular surface of both receptors such that it sterically hinders arrestin binding. This would be consistent with a CCL2-induced conformational change altering the position of the Gα_i1_ (and presumably the rest of the heterotrimeric complex as well), therefore potentially enabling increased recruitment of β-arrestin2, not only to CCR2, but also to AT_1_ receptor. This would be consistent with the synergistic increase in receptor-arrestin BRET signal that we observe upon co-activation. At this stage, this is merely speculation, however, we believe it to be an intriguing hypothesis.

Furthermore, it is interesting that the superior effect of combined antagonist treatment observed in HEK293FT cells is consistent with our *in vivo* findings. The potential for the allostery of heteromerization impacting receptor function is now very well established [1], as is the concept of biased signalling whereby one pathway is influenced differently to another [35]. The current study is certainly consistent with this, considering the aforementioned affects on Gα_i1_ and β-arrestin2, but little apparent effect on IP_1_ signalling. Our observations with β-arrestin2 are of particular interest as this critical intracellular scaffolding protein is not only involved in GPCR desensitization/internalization, but also promotes G protein-independent signalling pathways via many GPCRs including AT_1_ receptor [55]. Therefore, these findings provide further evidence for the potential importance of receptor heteromerization involving AT_1_ receptor, as recently shown for the α_1D_ adrenoceptor [56], CB_1_ cannabinoid receptor [57] and angiotensin II receptor type 2 [36].

The STNx rat model of progressive CKD resembles the major hallmarks of chronic kidney injury in humans, developing secondary hypertension, persistent proteinuria and declining GFR in conjunction with interstitial macrophage infiltration, depletion of podocytes, glomerulosclerosis and tubulointerstitial fibrosis. Proteinuria associated with glomerular podocyte loss has long been accepted as the clinical hallmark of progressive CKD [58], with a correlation between proteinuria and extent of podocyte loss being observed in patients and animal intervention studies [59,60]. In the present study, we demonstrated that the combination of Irb and PPG is superior to Irb monotherapy in attenuating podocyte loss and proteinuria. These outcomes appear to be independent of the blood pressure lowering effect of Irb. Previous studies have shown that CCR2 expression is greatly enhanced in glomerular podocytes of patients with CKD, with a positive correlation between CCR2 expression and extent of proteinuria [28]. Furthermore, in cultured podocytes, recombinant CCL2 induces apoptosis and conversely, inhibition of CCR2 is associated with a significant decrease in podocyte apoptosis [17]. AngII has been shown to cause podocyte apoptosis via AT_1_ receptor both *in vitro* and *in vivo* [18,61]. AngII, on the other hand, has also been demonstrated to induce CCL2 expression in renal tissues [62]. We speculate that AT_1_ receptor and CCR2 may act synergistically in mediating podocyte apoptosis, potentially as a receptor heteromer, and therefore blockade of both receptors is superior to inhibiting AT_1_ receptor or CCR2 alone.

In both inflammatory and non-inflammatory renal disease, macrophage infiltration is a prominent feature [47,63,64] and indeed, the number of interstitial mononuclear cells also correlates closely with declining renal function in a range of renal diseases [65]. These inflammatory cells, which contain reactive oxygen intermediates, proteases and inflammatory cytokines including CCL2, are viewed as playing a significant role in mediating cell apoptosis, proteinuria and fibrosis [63]. In the present study, interstitial macrophage infiltration was a prominent feature in the STNx rats. Treatment with PPG in combination with Irb was associated with a further reduction in macrophage infiltration when compared to PPG and Irb monotherapy. Thus, the further reduction in macrophage accumulation by blockade of both AT_1_ receptor and CCR2 may potentially contribute to the observed attenuation of podocyte loss and proteinuria.

In conclusion, our *in vivo* findings have demonstrated that combined inhibition of AT_1_ receptor and CCR2 signalling significantly reduces proteinuria, macrophage infiltration and podocyte loss, all of which are implicated in the pathogenesis of CKD. Furthermore, our novel GPCR-HIT assay approach has provided new insights into potential mechanisms of action that may contribute to this beneficial synergistic effect.
